# High-Energy Blast Injury Requiring Transradial Amputation with Associated Essex Lopresti and Terrible Triad Injuries of the Elbow: A Rare Presentation

**DOI:** 10.1155/2021/6645082

**Published:** 2021-03-03

**Authors:** Paul Knapp, Dexter Powell, Ivan Bandovic, Matthew Coon, Benjamin Best

**Affiliations:** ^1^Ascension Macomb-Oakland Hospital, 27351 Dequindre Rd, Madison Heights, MI 48071, USA; ^2^Ascension Providence Hospital, 16001 W Nine Mile Rd, Southfield, MI 48075, USA; ^3^Ascension St. John Hospital and Medical Center, 22101 Moross Road, Detroit, MI 48236, USA

## Abstract

**Case:**

Blast injuries to the upper extremity can be devastating and emotionally stressful injuries. We describe a case of a high-energy blast injury to an upper extremity from an explosive. The transfer of energy caused severe soft tissue/bony damage to the hand, but also led to associated Essex Lopresti and terrible triad injuries. The patient required emergent transradial amputation by hand surgery as well as definitive fixation by our orthopaedic team.

**Conclusion:**

We describe a unique salvage operation that established forearm pronosupination, elbow flexion, and proper prosthetic fitting. We feel that describing our technique could help others in treating this injury if encountered.

## 1. Introduction

The terrible triad injury of the elbow is a complex elbow dislocation with radial head and coronoid process fractures [1]. These injuries alone are difficult to manage and historically have poor outcomes, although more recent reports have provided evidence of good or excellent outcomes [2]. Restoration of the radiocapitellar articulation can be achieved by plate and screw fixation of the radial head or with radial head arthroplasty if the fracture is severely comminuted. Radial head excision has been shown to lead to inferior outcomes [3, 4] as the radial head is essential for valgus stability of the elbow, especially in cases with injury to the medial collateral ligament (MCL) [5].

In the original paper by Essex and Lopresti in 1951, the authors described an injury pattern involving “violent longitudinal compression” that causes a proximally directed force on the radius causing disruption of the interosseous membrane (IM) with an associated radial head fracture [3]. In a large majority of these cases, this is caused by a fall on an outstretched hand with a resulting axial load. Our patient had a combination of multiple commonly reported injuries including an Essex Lopresti injury, terrible triad injury pattern, and blast injury to the hand. The transfer of energy caused severe soft tissue/bony damage to the hand, but also associated Essex Lopresti and terrible triad injuries more proximal. The combination of all of these injuries is what makes this case very unique. We were unable to find any case reports in the literature describing this scenario.

## 2. Case Report

**Figure 1:**
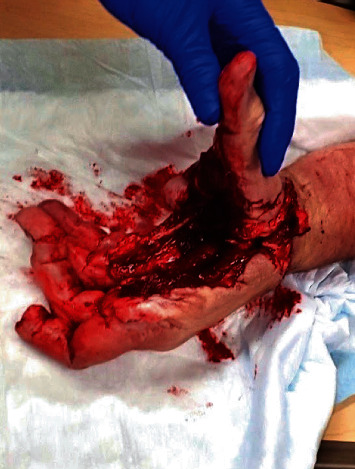


**Figure 2:**
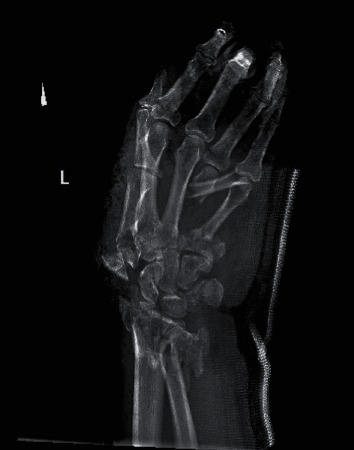


**Figure 3:**
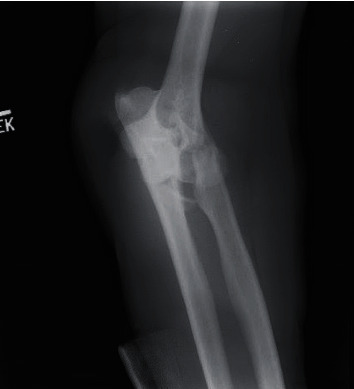


A 60-year-old right hand dominant male suffered a blast injury from a stick of dynamite. He was complaining of left hand, forearm, and elbow pain. Patient also endorsed significant numbness and tingling in the left hand. On physical exam, there was significant degloving of the palm and partial amputations of the second and third distal phalanges. Radial pulses were intact, with no evidence of an arterial bleed. A photograph of the hand on presentation can be seen in Figure 1. Figures 2 and 3 are the initial radiographs of the hand and elbow, respectively, showing the extent of the injury.

The patient went for emergent washout and exploration by hand surgery. He was found to have complete obliteration of the superficial and deep palmar arches. Approximately half of the flexor tendons were intact. The thumb was attached via a single flexor tendon and remnant skin. The median nerve was completely severed at the level of the wrist. Open reduction and internal fixation (ORIF) of the distal radius and the distal ulna was not performed because of a lack of viable soft tissue coverage secondary to the traumatic injury from the blast effect as determined by the hand surgeon intraoperatively. The amputation level was partially based on the level at which viable soft tissue coverage was available for coverage of bone. The hand surgeon proceeded with a transradial amputation.

**Figure 4:**
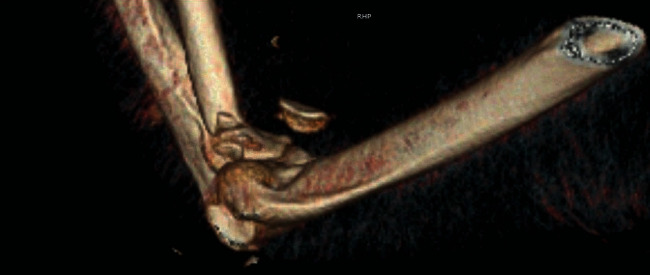


**Figure 5:**
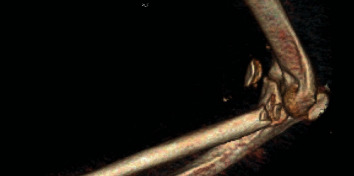


Closed reduction of the ulnohumeral joint was performed by the orthopaedic team once hand surgery was done with their part of the procedure. The patient required 90 degrees of flexion at the elbow to maintain reduction due to gross instability. 3D reconstructions from the postreduction computerized tomography (CT) scan are seen in Figures 4 and 5 showing the terrible triad injury of the elbow.

Twelve days after the initial injury, the patient returned to the operating room for definitive fixation. A Kaplan approach was used, which demonstrated rupture of the lateral collateral ligament (LCL) as well as a comminuted radial head fracture. An ulnar nerve decompression was performed because of preoperative ulnar nerve symptoms. A Hotchkiss “over the top” approach was used to expose the coronoid and medial collateral ligament (MCL), which demonstrated a midsubstance disruption. MCL was reconstructed and reattached to the medial epicondyle. The small coronoid tip fragment was excised. A cemented radial head arthroplasty was performed. LCL was then repaired. The remnant radial shaft and ulnar shaft were percutaneously pinned in neutral pronosupination due to instability noted intraoperatively.

**Figure 6:**
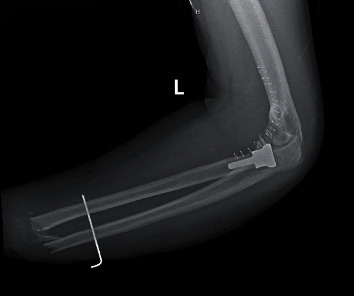


**Figure 7:**
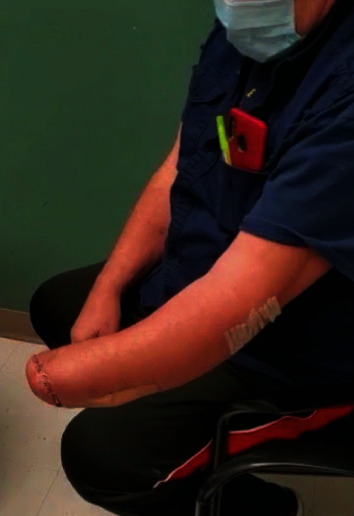


**Figure 8:**
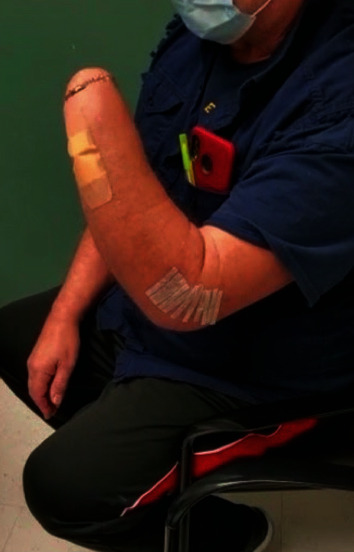


**Figure 9:**
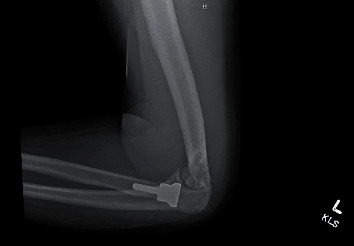


The patient was seen in the clinic two weeks postoperatively. Radiograph (Figure 6) revealed maintained reduction of the ulnohumeral joint and satisfactory alignment of the radial head replacement. The patient was noted to have a stable arc of motion between 20 and 90 degrees. Five weeks after reconstruction, the distal forearm pin was removed. The patient had a flexion arc of 5-90 degrees, but limited pronosupination. Two months postoperatively, his arc of motion was 5-115 degrees, as seen in Figures 7 and 8. He was able to attain 50 degrees of pronation and 50 degrees of supination. Radiographs at that time once again show intact hardware, seen in Figure 9.

**Figure 10:**
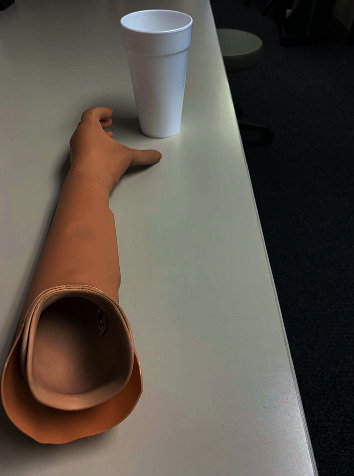


**Figure 11:**
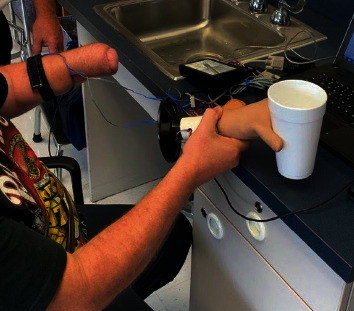


**Figure 12:**
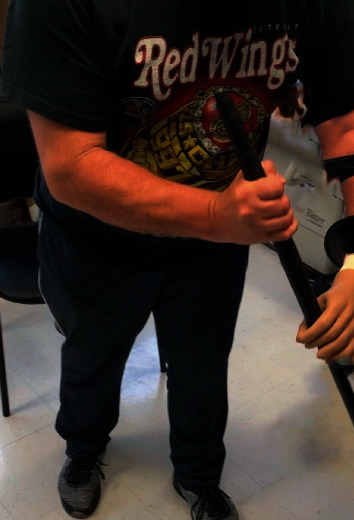


The patient was seen again at 12 months postop for prosthetic consult. The patient was fitted for prosthetic devices including the body-controlled hook prosthesis and the myoelectric hand prosthesis. The patient chose the myoelectric device due to the cosmetic appearance and fine motor capabilities.  Figure 10 shows the myoelectric device prior to fitting. Figures 11 and 12 show the patient during his prosthetic fitting. We will continue to watch his progress and long-term outcome.

## 3. Discussion

A main goal in upper extremity blast injuries is to achieve optimal functional outcomes, which depend on maintaining intact musculotendinous units, obtaining adequate range of motion, and achieving successful soft tissue coverage/cosmetic appearances [6]. Although the blast led to a forearm amputation, we were able to maintain a significant portion of his native function.

Transradial amputations, such as this case, are the most common upper extremity amputations [7]. The level of amputation is critical for maintenance of pronosupination. A “long,” or more distal, amputation can maintain up to 100-120 degrees of overall rotation, while a “short” amputation can provide anywhere from 0 to 60 degrees [8]. If the damage is too extensive and the chosen amputation is proximal to the elbow, the tremendous increase in the amount of work required to function the prosthetic limb has been shown to lead to noncompliance with the prosthetic limb [9].

Data has supported that a longer residual limb oftentimes leads to superior outcomes due to an increased ability of the patient to position the limb in space [10]. Our patient was a truck driver so this was essential to his outcome as we wanted to maintain length so that the stump could reach the wheel if he decided against a prosthetic. At his most recent clinical follow-up, the patient was hopeful that with his prosthetic he would be able to keep his job, instead of ending up on permanent disability. Literature shows that the majority of amputation patients do return to work, and the rates of returning to work are significantly higher in transradial amputations than more proximal amputations [7]. It has also been shown that transradial amputations have high rates of prosthetic utilization when compared with amputations above the elbow [7, 11].

Prosthetic options for transradial amputations typically include a body-controlled hook and varieties of myoelectric devices [10]. In recent years, there has been progress in myoelectric designs. Newer designs have used lighter materials, more efficient motors, and hydraulically controlled fingers which have led to a more accurate, anthropomorphic hand [12]. Our patient was enthusiastic about the myoelectric prosthetic and the fine motor skills it provided. The authors hope that he is able to use the prosthetic to return to work and activities of daily living.

In our review, we did find a case report of a rare traumatic divergent elbow dislocation with associated forearm amputation in an adult in an auger-related injury. In their reported case, the patient was treated with excision of the entire radius after reduction of the ulna. This determination was made due to the extensive stripping of the radial shaft and the inability to locate the median, radial, or ulnar nerves during their thorough exploration of the forearm. In transferring the distal biceps to the coronoid process, the authors were hoping to enhance elbow flexion [13].

In our case, we determined that there would be a benefit to maintaining the radial head articulation with a prosthetic head in order to assist with elbow stability but also allow for effective pronation and supination. Removal or excision of the proximal radial shaft/radial head would leave the patient with an inherently unstable elbow in the setting of a terrible triad injury as the radial head serves as a secondary stabilizer to valgus stress and helps to prevent dislocation. The modular radial head used in this case offers an efficient, practical solution for treating degenerative or posttraumatic conditions of the proximal radial head/neck. Its modular design means that the head and stem can be selected independently, allowing the head to closely match the diameter of the articulating surface of the native radial head while ensuring optimal stem fixation. After the stem is implanted, the head is loaded from the side, making it easier to assemble the implant in a tight joint. A set screw secures the head to the stem. The system can be used for primary and revision joint arthroplasty with cemented or uncemented press-fit applications. This is the most commonly used radial head prosthesis at our institution.

In our case, we believe that the treatment algorithm followed was the best option for this patient. Providing valgus stability and length with the prosthetic radial head was the first step followed by the collateral ligament repair/reconstruction. Once the elbow was deemed stable, we moved our attention to the distal end of the radius and ulna. We aimed to maintain as much length as possible. Pinning of the distal extent of the forearm was performed in order to create a stable stump for the prosthetic fitting. It is important to approach the patient and his/her history on a case by case basis. We hope that this case provides valuable information to the orthopaedic literature regarding treatment options for a unique injury pattern.

## Data Availability

As this is a case report, there is no specific data that was collected other than clinical examination findings and photos.
